# Computational analyses of obesity associated loci generated by genome-wide association studies

**DOI:** 10.1371/journal.pone.0199987

**Published:** 2018-07-02

**Authors:** Mengrong Cheng, Bing Mei, Qian Zhou, Manling Zhang, Han Huang, Lanchun Han, Qingyang Huang

**Affiliations:** College of Life Sciences, Central China Normal University, Wuhan, Hubei, China; University of North Carolina at Chapel Hill, UNITED STATES

## Abstract

**Objectives:**

Genome-wide association studies (GWASs) have discovered associations of numerous SNPs and genes with obesity. However, the underlying molecular mechanisms through which these SNPs and genes affect the predisposition to obesity remain not fully understood. Aims of our study are to comprehensively characterize obesity GWAS SNPs and genes through computational approaches.

**Methods:**

For obesity GWAS identified SNPs, functional annotation, effects on miRNAs binding and impact on protein phosphorylation were performed via RegulomeDB and 3DSNP, miRNASNP, and the PhosSNP 1.0 database, respectively. For obesity associated genes, protein-protein interaction network construction, gene ontology and pathway enrichment analyses were performed by STRING, PANTHER and STRING, respectively.

**Results:**

A total of 445 SNPs are significantly associated with obesity related phenotypes at threshold *P* < 5×10^−8^. A number of SNPs were eQTLs for obesity associated genes, some SNPs located at binding sites of obesity related transcription factors. SNPs that might affect miRNAs binding and protein phosphorylation were identified. Protein-protein interaction network analysis identified the highly-interconnected “hub” genes. Obesity associated genes mainly involved in metabolic process and catalytic activity, and significantly enriched in 15 signal pathways.

**Conclusions:**

Our results provided the targets for follow-up experimental testing and further shed new light on obesity pathophysiology.

## Introduction

Obesity is a worldwide epidemic with increasing global morbidity and mortality [1]. Obesity rates in adults aged 18+ have risen from around 9% in 1975 to 26% in 2014 with a marked increase over the past 4 decades [2]. In 2013, the American Medical Association (AMA) called obesity a disease, highlighting the importance of obesity in public health concern. Obesity results from a prolonged energy imbalance owing to intake exceeds expenditure, which may be defined as a condition in which there is an excessive accumulation of body fat and abnormally high body fat percentage caused by an increase in the number and volume of adipocytes at the cellular level. Obesity is a complex and multi-factorial disorder, defined by body mass index (BMI). Clinically, obesity is often accompanied by diversely serious chronic diseases, such as metabolic syndrome, type 2 diabetes (T2D), cardiovascular disease and certain forms of cancer, which become the leading causes of morbidity and mortality.

In the genome era, genome-wide association study (GWAS) is a major and effective approach to discover genes and SNPs that contribute to complex diseases. To date, 46 obesity GWASs and 12 meta-analyses had reported 445 SNPs involved in 389 genes with a significant threshold *P* < 5×10^−8^. The future challenge of obesity genetic study is to elucidate functional mechanisms through which these GWAS associated loci modulate obesity risk.

Computational approaches are powerful and essential means for post-GWAS studies, which can screen the potential and promising SNPs that deserve experimental testing for follow-up functional assays within amounts of candidate variants. Computational analyses represent a starting point to guide the functional research. Our group has previously characterized the GWAS SNPs and genes by computational approaches for osteoporosis [3], T2D [4] and Alzheimer's disease [5]. Here, we used computational approaches to comprehensively analyze obesity GWAS identified SNPs and genes, including functional annotation using RegulomeDB and 3DSNP, effects on miRNAs binding and protein phosphorylation for obesity associated SNPs, and protein-protein interaction network, gene ontology and pathway enrichment analyses for obesity associated genes, in order to identify functional SNPs for follow-up experimental assays, and to provide guidance for future study with regard to the pathogenesis and etiology of obesity.

## Methods

### Search strategy and data collection

**Fig 1:**
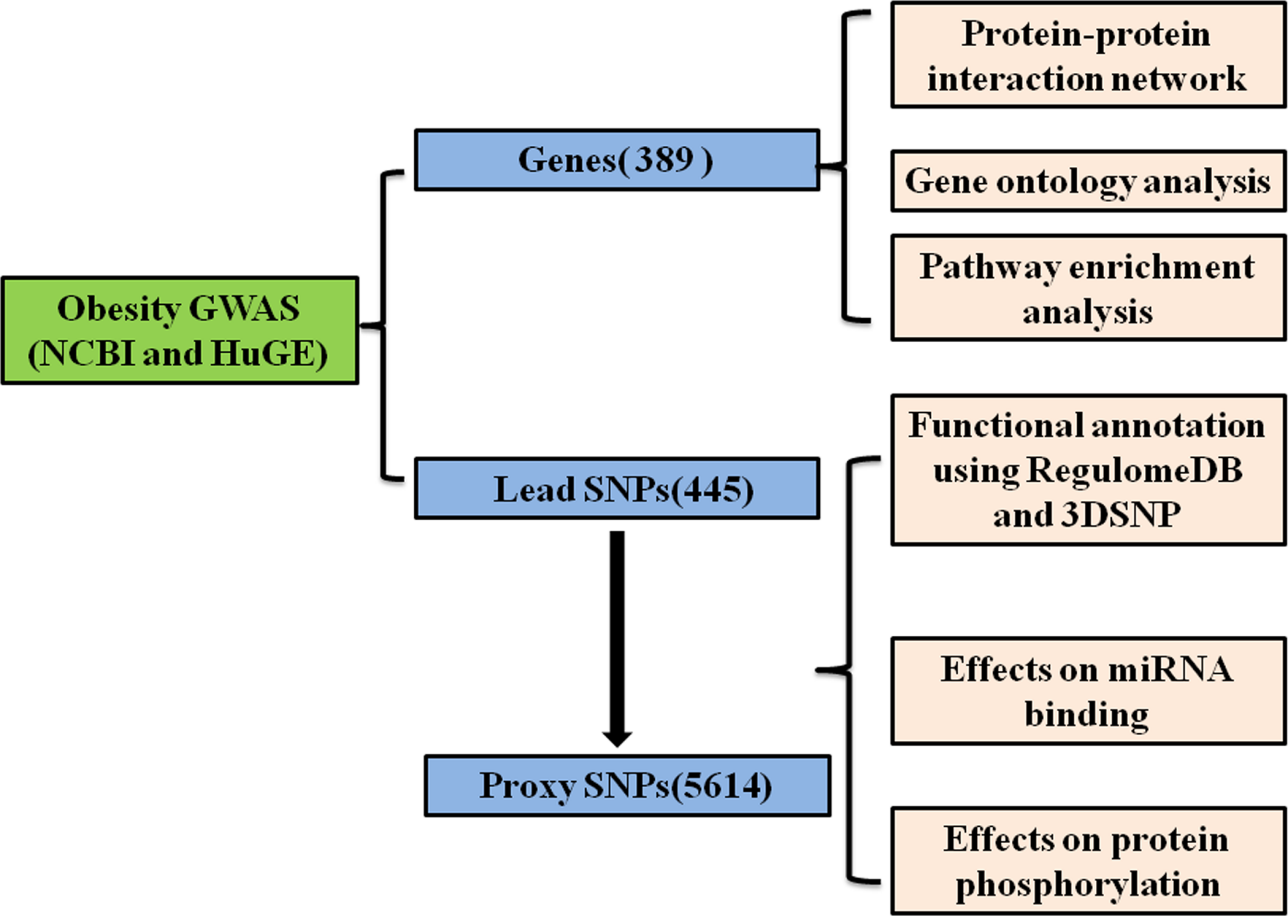
The workflow of obesity associated loci derived from GWAS. Data were obtained from the NCBI Association Results Browser (http://www.ncbi.nlm.nih.gov/projects/gapplusprev/sgap_plus.htm) and HuGE Navigator (http://64.29.163.162:8080/HuGENavigator/startPagePhenoPedia.do) and analyzed by many tools.

NCBI Association Results Browser and HuGE Navigator were used to extract obesity GWAS SNPs. “Obesity”, “BMI”, “body weight”, “overweight” and “FBM” were used as keywords and *P* < 5×10^−8^ as a significant threshold (updated to September, 2017). We first mapped these GWAS SNPs to the genome (hg19: GRCh37) by dbSNP (https://www.ncbi.nlm.nih.gov/snp/). Genes within a distance limit of 1Mb from GWAS SNPs were considered to be obesity GWAS genes, which were used to perform protein-protein interaction network, GO and KEGG pathway enrichment analyses. SNP Annotation and Proxy Search (SNAP, http://www.broadinstitute.org/mpg/snap/ldsearch.php) was used to identify proxy SNPs that were in strong linkage disequilibrium (LD) with obesity GWAS identified lead SNPs, based on genotype data from the 1000 Genomes Pilot 1 Project and the International HapMap Project (v3) with the CEU population panel. A distance limit of 500kb from the query lead SNP and r^2^ ≥ 0.8 from pairwise LD calculations were used as search and inclusion criteria. All loci were organized into an excel.file (S1 Table). A detailed workflow overview is illustrated in Fig 1.

### Functional annotation of obesity GWAS identified lead SNPs and their proxy SNPs via RegulomeDB and 3DSNP

RegulomeDB is an online database that provides functional interpretation of SNPs outside the coding region using high-throughput data from the ENCODE Project (ENCODE Project Consortium, 2012) and other resources like expression Quantitative Trait Loci (eQTL) and NCBI Sequence Read Archive. It is also a valuable tool for investigation of potential regulatory functions of SNPs on gene expression and disease phenotype. In RegulomeDB, SNPs are classified into classes based on the combinatorial presence/absence status of functional categories including protein binding, motifs, chromatin structure, eQTLs, histone modifications, and related data. And each SNP was assigned to an annotation score (range 1–7) to indicate the potential function [6]. 3DSNP is an integrated database for comprehensively annotating the regulatory function of human non-coding SNPs by exploring their three-dimensional (3D) interactions with genes and other genetically associated SNPs mediated by chromatin loops. Unlike the scoring scheme of RegulomeDB, 3DSNP integrates six different functional categories, including 3D interacting genes, enhancer state, promoter state, transcription factor binding sites, sequence motifs altered and conservation, in a scoring system to quantitatively evaluate the functionality of a SNP [7], and the sum of scores of the six functional categories is the total functionality score of a SNP. The higher 3DSNP score indicates the more likely functionality of a SNP. Here, we used RegulomeDB (v1.1) and 3DSNP (v1.0) to score each SNP.

### Effects of obesity GWAS identified lead SNPs and their proxy SNPs on miRNA binding

MiRNAs can modulate gene expression by predominantly decreasing mRNA stability through base pairing with the 3'UTR of target mRNAs. A small complementary sequence from 2 to 7 nucleotides long usually is involved in the recognition of target mRNA by miRNA. Thus, only one nucleotide alteration in recognition sequence by SNPs can either create or destroy miRNA binding sites, and increase or decrease the miRNA binding affinity with target mRNAs. Several online web-based databases, such as SNPinfo (http://snpinfo.niehs.nih.gov/cgi-bin/snpinfo/snpfunc.cgi), miRNASNP (http://bioinfo.life.hust.edu.cn/miRNASNP2/), mrSNP (http://mrsnp.osu.edu/), MirSNP (http://202.38.126.151/hmdd/mirsnp/search/), and PolymiRTS (http://compbio.uthsc.edu/miRSNP/), can provide a resource for identifying the miRNA-related SNPs. We used miRNASNP (v2.0) to predict the “gain or loss” effects of miRNA binding sites by SNPs in 3'UTR of target mRNAs.

### Effects of obesity GWAS lead SNPs and their proxy SNPs on protein phosphorylation

Protein phosphorylation is one of the most important reversible and intensively studied post-translational modification types, related to diverse signaling pathways and performing essential roles in modulating almost all kinds of biological processes and normal cellular functions. Protein phosphorylation is catalyzed by protein kinases (PKs) and principally targeting on serine (S), threonine (T), and tyrosine (Y) residues. In human genome, approximately 70% of nonsynonymous SNPs are potential phosphorylation-related SNPs (phosSNPs) that might influence protein phosphorylation status. Here, the PhosSNP 1.0 database was applied to identify phosSNPs for obesity GWAS lead SNPs and their proxy SNPs.

### Protein-protein interaction network analyses

Search Tool for the Retrieval of Interacting Genes (STRING, v10.0, http://string-db.org) is a database that covers known and predicted protein interactions, including direct (physical) and indirect (functional) associations. The database quantitatively integrates interaction data derived from four sources: genomic context, high-throughput experiments, co-expression (conserved) and previous knowledge. Currently, STRING covers 5,214,234 proteins from 1133 organisms. In this study, STRING was applied to build protein-protein interaction network.

### GO and pathway enrichment analyses

Gene ontology (GO, http://geneontology.org) is a widely utilized source of gene functional annotation including biological process, molecular function and cellular component. The Database for Annotation, Visualization and Integrated Discovery (DAVID, v6.8) (https://david.ncifcrf.gov/) was used to identify significantly enriched GO terms. Kyoto Encyclopedia of Genes and Genomes (KEGG) is a database resource for understanding high-level functions and utilities of the biological system. In our work, STRING was used to investigate the enriched KEGG pathways for obesity GWAS genes. KEGG pathways with FDR < 0.05 were considered to be significant.

## Results

### Obesity GWAS SNPs/genes

A total of 445 SNPs were identified to be associated with obesity, BMI, body weight, overweight or FBM at a significant threshold *P* < 5×10^−8^, and 389 genes were considered as obesity GWAS genes. Of these SNPs, 263 mapped to intronic regions, 63 mapped to intergenic regions, 64 located in gene upstream and 58 in gene downstream, 19 located in 3'UTR and 8 in 5'UTR, 15 missense variant, 10 nc transcript variants, and 3 synonymous variant. A total of 5,614 proxy SNPs were considered to be in strong LD with obesity GWAS lead SNPs by SNAP (r^2^ ≥ 0.80). The detailed information of obesity GWAS loci was showed in S1 Table.

### Functional annotation of obesity GWAS lead SNPs and proxy SNPs

Of the 6,059 SNPs, 36 SNPs returned error and 1,739 SNPs had score of ‘7’ that means no data available for these SNPs in RegulomeDB database, 4,284 SNPs returned with scores of 1–6, in which 680 SNPs (55 lead SNPs and 625 proxy SNPs) had score less than or equal to 3. The detailed regulatory functions of all GWAS SNPs and their proxy SNPs were given in S1 Table. Of particular note, four proxy SNPs which were in strong LD with obesity GWAS lead SNP rs977747 (located at 1p32 region) had scores of 1, were eQTLs for *PDZK1IP1*. Twenty-two proxy SNPs located at 1q21-q22 region had scores of 1, were eQTLs for *CTSS*. Four lead SNPs and multiple proxy SNPs located in 2p23.3 region were eQTLs for *ADCY3*. Additionally, some SNPs were located at the binding sites of obesity related transcription factors CEBPB, TCF7L2, STAT3, SPI1, GATA2, CREB1 and MEF2C (Table 1 and S2 Table). For 3DSNP, eleven GWAS lead SNPs had scores more than 100, and twelve GWAS lead SNPs were in the range of 60–100 (S1 and S3 Tables). GWAS lead SNP rs823114 had the highest 3DSNP score of 205.28 and a RegulomeDB score of 4, which located in 2kb upstream of the *NUCKS1* gene. RegulomeDB suggested that rs823114 influence the binding of 59 different proteins (S3 Table). GWAS lead SNP rs329120 had the second highest 3DSNP score of 204.84 and a RegulomeDB score of 4, which located in the intronic region of the *JADE2* gene and 500kb downstream of the *LOC107986451* gene. RegulomeDB uncovered that rs329120 affect the binding of 34 different proteins (S3 Table). The functions of *NUCKS1*, *JADE2* and *LOC107986451* in obesity are unknown, and need to explore in the future.

**Table 1:** SNPs located in the binding sites of obesity related transcription factors.

Transcription factor	SNPs
CEBPB	rs2815752,rs4836133,rs17150703,rs11191580,rs7132908,rs9925964,rs329120,rs2270204,rs11257655
TCF7L2	rs11208659,rs6548238,rs13201877,rs7132908,rs329120,rs823114,rs2357760,rs427943
STAT3	rs4836133,rs7132908,rs9925964,rs9940128,rs1861866,rs11671664,rs329120,rs2281727, rs4430979
SPI1	rs13191362,rs1121980,rs11671664,rs329120,rs633715,rs11257655,rs3783890
GATA2	rs2272903,rs9400239,rs7132908,rs4357030,rs11257655
CREB1	rs823114

### Effects of obesity GWAS lead SNPs and proxy SNPs on miRNA binding and protein phosphorylation

Using miRNASNP, nine obesity GWAS lead SNPs and 35 proxy SNPs might influence the recognition and targeting of miRNAs (S4 Table). Among these miRNAs, miR-326, let-7, miR-31, miR-342, miR-181a, miR-148, miR-196a and miR-548 have been reported to relate with adipogenesis and lipid metabolism. The target genes of these miRNAs were further identified using Target Scan Human 7.1 and literature mining through NCBI PubMed (Table 2).

**Table 2:** The target genes of the miRNAs reported to relate with adipogenesis and lipid metabolism.

miRNA	Predicted and validated target genes
miR-326	*RASSF1*, *AAK1*, *FAIM2*, *DGKG*, *ARAP1*, *TCF4*,
miR-31	*PIK3C2A*, *C/EBPα*, *IDE*, *FTO*, *SLC2A4*
miR-548d-5p	***PPARγ*,** *ADAM30*, *NFKB1*, *LIN7C*, *CDKN2B*, *KRAS*, *TP53INP1*, *ABCB5*, *HHEX*, *PIK3C2A*, *SLC30A8*, *PTEN*, *EXOC4*, *JAZF1*, *VPS26A*, *ADIPOQ*, *IRS1*, *CCDC171*, *TMEM18*, *G6PC2*,
miR-342	***CTBP2***, *CCDC171*, *EP300*, *SH2B1*
let-7	***HMGA2***, *ADCY9*, *IGF2BP2*, *KCNJ11*, *KCTD15*, *SEC16B*
miR-196a	***HOXC8***, *ACACB*, *ADIPOQ*,* CCDC171*, *G6PC2*, *NEGR1*
miR-181a	***TNF-a***, ***Smad7***,***Tcf7l2***, ***IDH1***,***sirtuin1 (SIRT1)***, *CDKN2B*, *FAM120A*, *SLC30A8*
miR-148	***WNT1***, *ADCY9*, *ASB4*, *CREB1*, *FAM120A*, *FAM120AOS*, *IRS1*, *KCTD15*, *LIN7C*, *LPL*, *NEGR1*, *NOTCH2*, *SLC30A8*, *KLF9*

Note: Bold genes represent validated target genes of miRNAs.

Ten obesity GWAS lead SNPs and thirteen proxy SNPs may affect protein phosphorylation (Table 3). Among them, SNP rs6265 within *BDNF* influencing BDNF phosphorylation has been verified [8].

**Table 3:** Effect of obesity lead SNPs and proxy SNPs on protein phosphorylation.

SNPs		Mapped genes	Types
**Lead SNPs**	rs11676272	*ADCY3*	Type I(−), Type II(−)
	rs6265	*BDNF*	Type II(−)
	rs591120	*SEC16B*	Type III(+)/(−)
	rs671	*ALDH2*	Type II(+),Type III(−)
	rs7498665	*SH2B1*	Type I(+)
	rs2230061	*CTSS*	Type III(+)
	rs1190736	*GPR101*	Type II (+)/(−),Type III(+)/(−)
	rs2228213	*HIVEP1*	Type II(−),Type III(+)/(−)
	rs5215	*KCNJ11*	Type III(−)
	rs17826219	*ATAD5*	Type III(+)/(−)
**Proxy SNPs**	rs749670	*ZNF646*	Type III(+)
	rs12102203	*DMXL2*	Type I(−), Type II(+), Type III(+)
	rs61750814	*NUP54*	Type I(+), Type III(+)/(−)
	rs2277598	*BBS4*	Type I(+), Type III(+)
	rs1344642	*STK36*	Type II(−), Type III(−)
	rs2230115	*ZNF142*	Type I(+), Type III(+)
	rs3770213	*ZNF142*	Type III(+)/(−)
	rs3770214	*ZNF142*	Type I(−)
	rs2228209	*HIVEP1*	Type II(+),Type III(+)/(−)
	rs3816780	*ATAD5*	Type I(+),Type III(+)/(−)
	rs11657270	*ATAD5*	Type I(−),Type II(−),
	rs9910051	*ATAD5*	Type III(+)/(−),Type IV(−)
	rs1336900	*HORMAD1*	Type I(−),Type III(+)

Note: Type I (+)/(−): change of an amino acid with S/T/Y residue or vice versa to create a new or remove an original phosphorylation site.

Type II (+)/(−): variations to add or remove adjacent phosphorylation sites.

Type III (+)/(−): mutations to change PK types of adjacent phosphorylation sites.

Type IV(+)/(−): an amino acid substitution among S, T, or Y that could change the PK types in the phosphorylated position.

### Protein-protein interaction network

**Fig 2:**
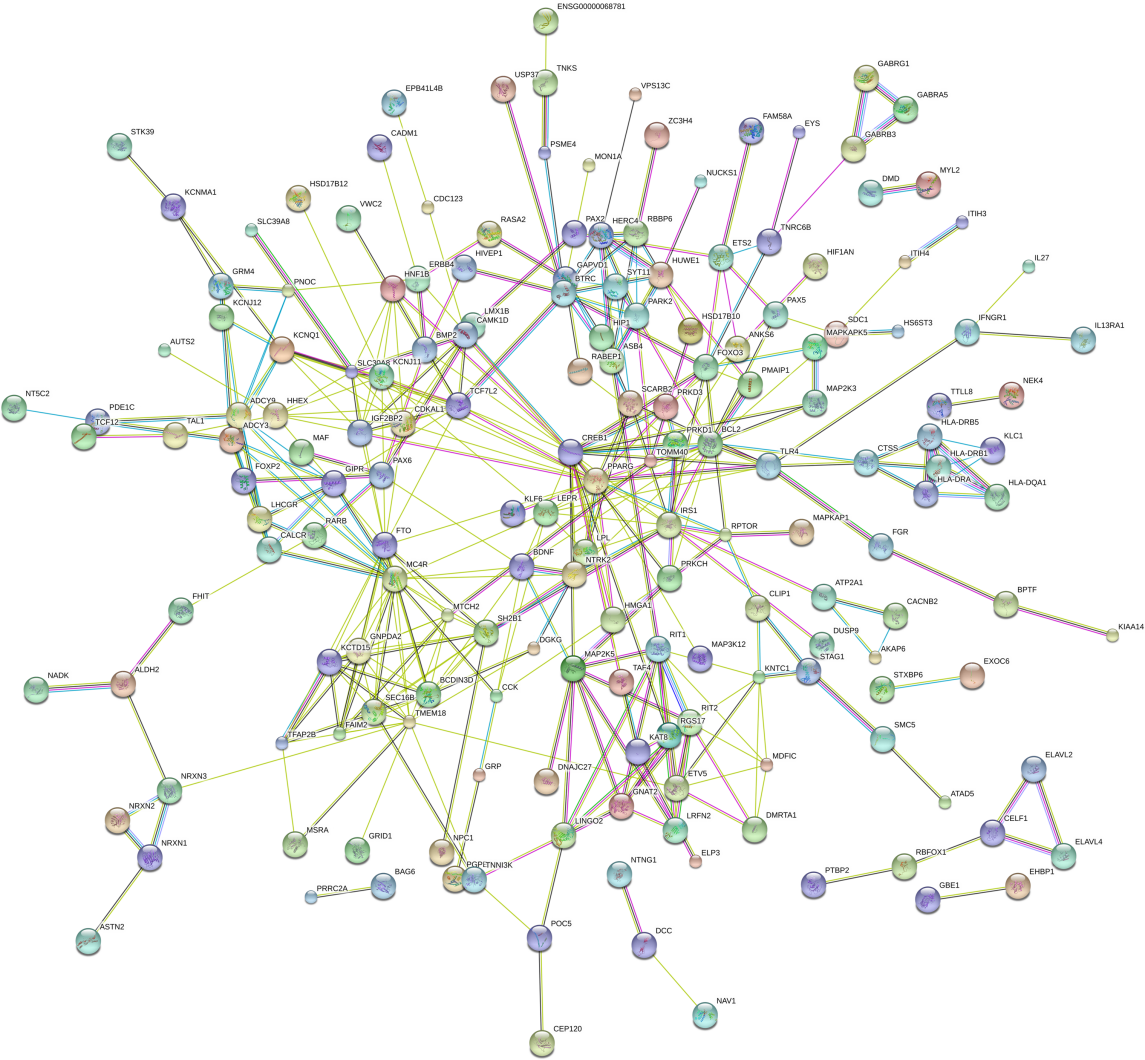
Protein-protein interaction network of obesity GWAS associated genes. The nodes and edges represent the proteins (genes) and their interactions, respectively. Colored nodes represent query proteins and first shell of interactors, white nodes represent second shell of interactors, empty nodes represent proteins of unknown 3D structure, filled nodes represent some 3D structure is known or predicted. Purple edges (experimentally determined) and light blue edges (from curated databases) represent known interactions, green edges (gene neighborhood), red edges (gene fusions) and dark blue edges (gene co-occurrence) represent predicted interactions, yellow-green edges (textmining), black edges (co-expression) and light blue edges (protein homology) represent others.

Fig 2 showed protein-protein interaction network of obesity GWAS genes coding proteins. Proteins with the strong connections were considered as being ‘Hub proteins’, such as CREB1, MC4R, PPARG, TMEM18, FTO, BCL2, TCF7L2, IRS1, SH2B1, KCNJ11, SEC16B, SLC30A8, BCDIN3D, BTRC, CDKAL1, FOXO3, GNPDA2, IGF2BP2, KCTD15, MTCH2 and RIT2 (S5 Table). These hub proteins were mainly involved in AMPK signaling pathway (CREB1, FOXO3, IRS1 and PPARG), Neurotrophin signaling pathway (BCL2, FOXO3, IRS1 and SH2B1), PI3K-Akt signaling pathway (BCL2, CREB1, FOXO3 and IRS1), Circadian rhythm (BTRC and CREB1), T2D (IRS1 and KCNJ11), and several cancer associated pathways. These hub proteins encoding genes were defined as “hub” genes.

### GO and pathway enrichment analyses of obesity GWAS genes

GO and pathway enrichment analyses found that obesity GWAS genes significantly enriched in 104 GO functional categories with *P*-value < 0.05 (S6 Table) and 15 KEGG signaling pathways with FDR < 0.05 (S7 Table). Notably, of 104 GO functional categories, two GO terms “glucose homeostasis” and “response to glucose” were also enriched by T2D GWAS genes (such as TCF7L2 and FTO), indicating that obesity associated genes might confer T2D risk through its primary effect on adiposity.

## Discussion

One of the molecular mechanisms by which SNPs mediate the onset of a disease is to regulate gene expression by affecting the binding of transcription factors, and increase disease susceptibility [9]. For example, SNP rs4684847, in LD with obesity GWAS lead SNP rs1801282, is located in the upstream 6.5 kb of *PPARG2* promoter. The risk C allele of rs4684847 could enhance the binding to PRRX1 and inhibit *PPARG2* mRNA expression, thereby leading to abnormal regulation of free fatty acids turnover and glucose homeostasis [10]. In this study, we identified some obesity GWAS SNPs that were located at the binding sites of adipogenic differentiation and lipid metabolism related transcription factors CEBPB, TCF7L2, STAT3, SPI1, GATA2, CREB1 and MEF2C, and were eQTLs of obesity or lipid metabolism associated genes, such as *ADCY3*, *CTSS*, *KCTD15*, *MTCH2* and *SPI1*. Genetic and epigenetic studies have demonstrated that *ADCY3* is involved in the pathogenesis of obesity [11]. ADCY3 haploinsufficiency resulted in increased expression of genes involved in adipogenesis in peripheral tissues of mice [12]. *CTSS* gene expression increases with obesity in the adipose tissue of obese rodent and human. Compared with normal-weight subjects, obese subjects had a 2-fold increase in *CTSS* mRNA in adipose tissue and 30% increase in circulating CTSS levels [13]. Variants of the *KCTD15* were associated with risk for obesity. In adipose tissues of 2 obesity mice models, *KCTD15* exhibited concomitant obesity-dependent alterations in DNA methylation and gene expression [14]. MTCH2 is a conserved regulator of lipid homeostasis. Knockdown of *MTCH2/mtch2* reduced lipid accumulation (in adipocyte-like cells, C. elegans and mice) [15] and number of adipocytes (in zebrafish) [16], while overexpression of *MTCH2* increased fat accumulation (in adipocyte-like cells, C. elegans and mice) [15,17]. Loss of muscle *MTCH2* protected mice from diet-induced obesity and hyperinsulinemia and increased energy expenditure [18]. The transcription factor SPI1 is expressed in mature adipocytes of white adipose tissue, whose mRNA and protein levels is increased markedly in mouse models of genetic or diet-induced obesity [19].

Another molecular mechanism by which SNPs mediate disease susceptibility is to affect the binding of miRNAs [9]. In this study, 9 lead SNPs and 35 proxy SNPs were predicted to affect the binding of miRNAs (S2 Table). Among them, of particular note, SNP rs7132908 located in the 3'UTR of *FAIM2* had a 3DSNP score of 162.69. The rs7132908 A allele was predicted to destroy the binding sites for hsa-miR-330-5p and hsa-miR-326. SNP rs2650492 located in the 3'UTR of *SBK1*, had a 3DSNP score of 90.2 and a RegulomeDB score of 2c. The rs2650492 A allele was predicted to destroy the binding sites for hsa-miR-331-3p. SNP rs2531995 located in the 3'UTR of *ADCY9*, had a 3DSNP score of 43.01 and a RegulomeDB score of 1f. The rs2531995 A allele was predicted to create the binding sites for hsa-miR-632 and hsa-miR-654-3p. Therefore, it is very likely that these SNPs play regulatory function by affecting miRNA binding, which needs to be confirmed by experimental methods in the future.

Abnormal protein phosphorylation has been reported in a number of diseases, such as Parkinson's disease, Alzheimer's disease, and other degenerative disorders. Deng et al. [8] identified and characterized phosSNPs significant for bone mineral density in humans, and successfully dissected the functions of phosSNPs. Niu et al. [20] showed that phosSNPs rs3755955 and rs6831280 of *IDUA*, rs2707466 of *WNT16* associate with bone mineral density phenotypes, and in silico analyses revealed that phosSNP rs2707466 of *WNT16* directly destroyed a phosphorylation site, which could have a deleterious effect on WNT16 protein, but phosSNPs rs3755955 and rs6831280 of *IDUA* might play indirect effects on nearby phosphorylation sites. In this study, 10 GWAS lead SNPs and 13 proxy SNPs were identified as being phosSNPs that might affect the protein phosphorylation status, indicating that similar mechanism might also exist in obesity.

Protein-protein interaction network identified a number of key “hub” genes including *FTO*, *TMEM18*, *MC4R*, *CREB1*, *PPARG* and *TCF7L2*. *FTO* was the first obesity susceptibility gene identified by GWASs and continued to be the locus with the largest effect on obesity risk and BMI, most widely replicated with variety of obesity traits across diverse ancestries and throughout the life course [21]. Intriguingly, in human brains, obesity associated SNPs within FTO are functionally connected, at megabase distances, with regulation of the homeobox gene *IRX3* expression, but not *FTO*, and *in vivo* studies in mice demonstrated that the expression levels of *IRX3* affect body mass and composition phenotypes, suggesting that although the obesity associated SNPs reside in the first intron of *FTO* gene, they may not only influence *FTO* but mediate their obesity effects through long-range interaction with nearby genes (notably *IRX3* and *RPGRIP1L*) [22]. Of note, as the first mRNA demethylase that has been identified, the demethylase activity of FTO is required for adipogenesis [23]. *TMEM18* is the second largest effect size among all loci identified so far via GWASs or GWAS meta-analyses [24]. *TMEM18* seems to influence energy levels via insulin and glucagon signaling, and significantly inhibited adipocyte maturation in human adipogenesis [25]. MC4R is widely expressed in the central nervous system, which plays an important role in the leptin-melanocortin pathway in modulating energy homeostasis, affecting both energy intake and expenditure [26]. In both *MC4R* knock-out mice and humans, mutations in *MC4R* could cause decreased energy expenditure and increased food intake [26,27]. CREB1 is a transcription factor that can drive the expression of a number of genes involved in the regulation of food intake and energy expenditure. Expression of constitutively active CREB induced expression of endogenous C/EBP β, and caused adipogenesis [28]. Mice that lack *CREB1* in SIM1-positive neurons developed an obese phenotype as a result of reduced energy expenditure, not because of excessive energy intake [29]. PPARG is a member of the nuclear receptor superfamily of ligand-dependent transcription factors and that functions as a master regulator of adipocyte differentiation and metabolism [30]. TCF7L2 is an important transcription factor in the canonical Wnt signaling pathway, and Wnt signaling via TCF7L2 can inhibit adipogenesis [31].

Pathway enrichment analysis identified 15 signaling pathways. Neurotrophins are a family of trophic factors, consisting of BDNF, nerve growth factor, neurotrophin 3, and neurotrophin 4, which exert their opposite functional outcomes through engagement of p75 neurotrophin receptor (p75^NTR^) or Trk tyrosine kinase receptors. Neurotrophin receptor signaling could affect how the central nervous system control body weight change and energy intake [32–34]. In the hypothalamus, BDNF signals through TrkB could suppress appetite and reduce body weight. Mice conditionally-depleted of *BDNF* in neurons [33] or *BDNF*^*+/-*^ mice [32] overeat and become obese on a normal diet. Adipocyte-specific deficiency of p75^NTR^ or transplantation of p75^NTR^-null white adipose tissue into wild-type could protect mice from high-fat diet-induced obesity **a**nd insulin resistance [35]. GWAS have identified associations between BMI and two loci close to cell adhesion molecule 1 and 2 (*CADM1* and *CADM2*), risk variants within them associate with elevated *CADM1* and *CADM2* expression in the hypothalamus of human subjects, respectively. In obese mice, expression of *CADM1* and *CADM2* increased, and *Cadm1* loss protected mice from obesity. In excitatory neurons, induction of *Cadm1* could facilitate weight gain while exacerbate energy expenditure. In the hypothalamus and hippocampus, decreased *Cadm1* expression promoted a negative energy balance and weight loss [36]. Approximately 15–40% of inflammatory bowel disease (IBD) patients are obese. IBD and obesity share environmental link and mechanistic connection, obesity can contribute to the development of IBD and response to therapy in IBD patients [37]. It is suggested that toxoplasmosis associate with obesity by alteration of inflammatory fat distribution as organisms change and reside in fatty tissues [38]. Men and women with cutaneous leishmaniasis presented higher body mass index than the controls [39]. Association between overweight and asthma have been found among females, persistent asthma associated with high BMI throughout childhood [40]. Also, BMI has been shown to be associated with the risk of immune-related infectious diseases such as tuberculosis [41]. Besides, high-fat diet-induced obesity can enhance allograft rejection [42]. Obesity is considered as a risk factor for posttransplantation complications including acute graft-versus-host disease [43].

Our computational analyses have some strengths and weaknesses. Compared with labour-intensive and time-consuming wet experiments, computational approach could quickly identify the promising and potential causal SNPs from a large amount of GWAS variants, and the validity has been proved by previous work [8,9]. On the other hand, each computational tool has its own unique features, and a single approach might not correctly find true causal SNP. Application of multiple methods would reduce false discovery rate. An appropriate follow-up to our study would be to validate computational prediction by wet experiments. This two-step process of identifying potential functional SNPs using computational tools followed by conventional experimentation would be a good strategy to reveal functional mechanisms at obesity GWAS loci.

## Conclusions

Our computational analysis identified a number of SNPs that located at binding sites of obesity related transcription factors, and were eQTLs for obesity associated genes, and might affect miRNAs binding and protein phosphorylation. Protein-protein interaction network analysis identified the highly-interconnected “hub” genes. Obesity associated genes significantly enriched in 104 GO categories and 15 signaling pathways. Taken together, our study uncovered potential functional mechanisms of obesity GWAS SNPs and genes, and provided targets and clues for future functional analysis with regard to the etiology and pathogenesis of obesity.

## Supporting information

S1 Table The detailed regulatory functions for obesity GWAS lead SNPs and the proxy SNPs. (XLSX)

S2 Table Proxy SNPs located in the binding sites of obesity related transcription factors. (DOCX)

S3 Table Eleven GWAS lead SNPs with 3DSNP score more than 100. (DOCX)

S4 Table Effect of lead and proxy SNPs on the binding of miRNAs (gain or loss). (DOCX)

S5 Table Hub genes in obesity associated genes and their interactions. (DOCX)

S6 Table Enriched GO categories of obesity GWAS genes. (XLSX)

S7 Table Enriched signaling pathways of obesity GWAS genes. (DOCX)
