# Supplementary material for: Computational analyses of obesity associated loci generated by genome-wide association studies
Source: PLoS One. 2018 Jul 2;13(7):e0199987. doi: 10.1371/journal.pone.0199987 (PMC6028139; doi:10.1371/journal.pone.0199987)
Supplement: S2 Table — (DOCX) [file pone.0199987.s002.docx]

**S2 Table. Proxy SNPs located in the binding sites of obesity related transcription factors**

| **Transcription factor** | **SNPs** |
| --- | --- |
| CEBPB | rs4970988,rs4970987,rs7567997,rs939583,rs6760543,rs13393304,rs56328583,rs10189479,rs524012,rs524902,rs67487119,rs11191479,rs4752999,rs11039290,rs2280231,rs11030107,rs11041993,rs11634999,rs4776987,rs56314408,rs29937,rs1014988,rs1029871,rs10509189,rs10514301,rs10518899,rs10740134,rs10761766,rs10793295,rs11191479,rs11191580,rs12446550,rs12586312,rs12603276,rs12899036,rs13393304,rs1412234,rs1470581,rs2158045,rs216172,rs2247560,rs2269916,rs2574717,rs2644119,rs2819347,rs359592,rs3820594,rs420158,rs4245459,rs4486511,rs450231,rs4684848,rs474513,rs4774902,rs4836133,rs539298,rs6001827,rs6001834,rs6038557,rs6054392,rs6061981,rs6545368,rs6739303,rs6746275,rs6749375,rs675162,rs6751993,rs6751994,rs6755502,rs6760543,rs7103411,rs7132908,rs7307735,rs7501939,rs7638903,rs7647481,rs7649970,rs823096,rs939583,rs9915139,rs999798 |
| TCF7L2 | rs4970988,rs12096831,rs7529194,rs4674308,rs41303827,rs12413046,rs10883832,rs2280231,rs17603116,rs4775966,rs7187776,rs62037367,rs2303222,rs2836751,rs10745532,rs10883832,rs11874362,rs12325113,rs12413046,rs12586312,rs13063160,rs1866268,rs1935949,rs2280406,rs2526754,rs2574720,rs3824754,rs4678433,rs4684848,rs474513,rs539298,rs548288,rs6001827,rs6444081,rs6548238,rs6739303,rs6744646,rs6744653,rs708611,rs7132908,rs7187776,rs7359509,rs7649970,rs8906,rs9369083,rs9891656,rs9891957,rs9968605,rs999798,rs9968605 |
| STAT3 | rs13393304,rs500317,rs500422,rs41303827,rs6485753,rs4752990,rs11030107,rs56133711,rs484983,rs2303222,rs29937,rs1029871,rs10852610,rs10852932,rs13393304,rs1582619,rs1866268,rs4659905,rs4684848,rs4836133,rs4854343,rs6444081,rs6739303,rs675162,rs708724,rs7132908,rs7359509,rs7649970,rs7737742,rs7939352,rs9940128,rs9940646,rs999798 |
| SPI1 | rs4970988,rs4970987,rs9631062,rs833083,rs10946403,rs9633712,rs7110437,rs7101471,rs10519313,rs7187776,rs889548,rs55731973,rs11130317,rs1121980,rs1187323,rs1231209,rs12325113,rs12355784,rs3733041,rs4546329,rs4659905,rs6475737,rs6475738,rs6617,rs708725,rs7187776,rs7359509,rs868117,rs9633712,rs9966656 |
| GATA2 | rs2984618,rs4970987,rs4970988,rs2867131,rs10189479,rs3731867,rs41303827,rs11070860,rs10519313,rs62037367,rs55731973,rs10412132,rs10518899,rs10733793,rs10761766,rs10793295,rs11607801,rs11874362,rs12956208,rs13266634,rs1563966,rs1788821,rs1893447,rs2393966,rs2510033,rs2526754,rs2644114,rs2867131,rs2893919,rs359592,rs3740331,rs3807648,rs3889747,rs4357030,rs4944195,rs6001834,rs6696888,rs6882366,rs708724,rs7132908,rs7231263,rs7244243,rs7939352,rs833752,rs901064,rs9398172,rs9400239,rs9651769,rs990706 |
| CREB1 | rs500422,rs2280231,rs7187776,rs7198606,rs11864750,rs55731973,rs999798,rs12325113,rs7187776,rs7198606,rs6617 |
| MEF2C | rs9818006,rs10946403,rs9915667,rs7220999,rs4245459,rs1473553 |
