# Supplementary material for: Computational analyses of obesity associated loci generated by genome-wide association studies
Source: PLoS One. 2018 Jul 2;13(7):e0199987. doi: 10.1371/journal.pone.0199987 (PMC6028139; doi:10.1371/journal.pone.0199987)
Supplement: S3 Table — (DOCX) [file pone.0199987.s003.docx]

**S3 Table. Eleven GWAS lead SNPs with 3DSNP score more than 100**

| **SNP** | **Nearest Gene** | **3DSNP Score** | **RegulomeDB Score** | **Bound protein** |
| --- | --- | --- | --- | --- |
| rs823114 | *NUCKS1* | 205.28 | 4 | CREBBP, CTCF, JUNB, MYC, TAF1, POLR2A, E2F4, E2F1, PML, SP4, TCF7L2, STAT5A, ATF2, GTF2F1, NFATC1, MAZ, SMARCC2, GABPB1, SIX5, STAT1, ATF3, REST, RUNX3, NFIC, TEAD4, TFAP2C, TRIM28, YY1, GATA1, HNF4A, TBP, NFKB1, BCLAF1, MAX, EP300, IRF4, MXI1, CHD2, EGR1, NFYA, TBL1XR1, ZBTB7A, ATF1, FOS, ARID3A, SP1, ZNF143, IRF3, IRF1, FOXM1, CREB1, NFYB, MYBL2, BHLHE40, JUND, SP2, NR3C1, CDX2, MTA3 |
| rs329120 | *JADE2/LOC107986451* | 204.84 | 4 | BHLHE40, CEBPB, CHD1, CHD2, CTCF, E2F6, ELF1, ETS1, GABPA, GATA1, GTF2F1, HDAC2, JUND, MAX, MAZ, MYC, PAX5, PHF8, POLR2A, RAD21, RCOR1, REST, SIN3A, SMC3, SPI1, STAT1, STAT3, STAT5A, TAF1, TBL1XR1, TCF7L2, UBTF, YY1, ZNF263 |
| rs80117551 | *HERC4* | 201.91 | 4 | POLR2A, TFAP2A, PHF8, RBBP5, CDX2, CHD1, SIN3A, GABPB1, MAX, MXI1, NFKB1, TAF1, HDAC1, SAP30, KDM5B, TBL1XR1, TFAP2C, IKZF1, EBF1, NFIC, PAX5, CCNT2, PML, FOXP2, YY1, MYC, IRF1, MAZ, ELK4, ETS1, EP300, GABPA, REST, RAD21, TAF7, TBP, TCF3 |
| rs7132908 | ***FAIM2*** | 162.69 | 4 | ATF1, BCLAF1, BRCA1, CBX3, CCNT2, CDX2, CEBPB, CHD2, E2F1, E2F4,E2F6, ELF1, ELK1, EP300, FOS, FOXP2, GABPB1, GATA1, GATA2, GATA3, GTF2F1, HDAC1, HDAC2, IRF1, JUN, JUNB, JUND, KDM5B, MAFK, MAX, MAZ, MXI1, MYC, NRF1, PML, POLR2A, RAD21, RBBP5, RCOR1, REST, RFX5, SIN3A, SMC3, STAT1, STAT3, STAT5A, TAL1, TBL1XR1, TBP, TCF7L2, TEAD4, TFAP2C, TRIM28, UBTF, USF1, USF2, YY1, ZKSCAN1, ZNF217, ZNF263 |
| rs9925964 | *KAT8* | 139.7 | 4 | CEBPB, FOS, MYC, NR3C1, POLR2A, STAT3 |
| rs11671664 | ***GIPR*** | 139.11 | 1a | ATF2, CCNT2, CHD1, CREBBP, CTBP2, CTCF, E2F1, EBF1, ELF1, EP300, EZH2, GABPB1, JUND, MAX, MAZ, MEF2A, NFKB1, NR2F2, PAX5, PHF8, PML, POLR2A, RAD21, RCOR1, REST, RUNX3, SIN3A, SPI1, STAT3, STAT5A, TBP, TCF12, TRIM28, USF1, WRNIP1, YY1, ZBTB7A, ZNF143, ZZZ3 |
| rs2365389 | *FHIT* | 126.32 | 4 | BHLHE40, E2F6, EBF1, MAX, MXI1, MYC, SIN3A, USF1, USF2 |
| rs2237897 | ***KCNQ1*** | 124 | 4 | BHLHE40, CTCF, HMGN3, HNF4A, JUND, MAX, MAZ, POLR2A, RAD21, SMC3, SP1, TFAP2A, TFAP2C, ZNF143 |
| rs17001654 | *SCARB2* | 116.67 | 4 | AR, EP300, FOS, FOSL2, FOXP2, JUND, MAX, MXI1, MYBL2, MYC, POLR2A, RXRA, SIN3A, TRIM28 |
| rs10838738 | ***MTCH2*** | 107.63 | 6 | - |
| rs12044597 | *NADK* | 106.86 | 5 | - |
| rs180950758 | *SUZ12P1* | 102.79 | 4 | BHLHE40, EGR1, MAX, USF1 |
| rs564343 | ***PACS1*** | 101.75 | 4 | AR, NR3C1 |

Note: Genes in bold have been reported previously to correlated with obesity.
