# Supplementary material for: Computational analyses of obesity associated loci generated by genome-wide association studies
Source: PLoS One. 2018 Jul 2;13(7):e0199987. doi: 10.1371/journal.pone.0199987 (PMC6028139; doi:10.1371/journal.pone.0199987)
Supplement: S4 Table — (DOCX) [file pone.0199987.s004.docx]

**S4 Table. Effect of lead and proxy SNPs on the binding of miRNAs (gain or loss)**

| SNPs | miRNA (loss) | miRNA (gain) |
| --- | --- | --- |
| ***Lead*** ***SNPs*** |  |  |
| rs3810291*/ ZC3H4*  (C→T) |  | hsa-miR-501-3p/hsa-miR-502-3p |
| rs2650492 /*SBK1*  (G→A) | hsa-miR-331-3p |  |
| rs10733682 /*LMX1B*  (A→G) |  | hsa-miR-940 |
| rs6465468/*ASB4*  (G→T) |  | hsa-miR-101-5p |
| rs879620/*ADCY9*  (G→A) | hsa-miR-892a |  |
| rs2531995/*ADCY9*  (G→A) |  | hsa-miR-632/ hsa-miR-654-3p |
| rs7132908/*FAIM2*  (G→A) | hsa-miR-330-5p**/hsa-miR-326** |  |
| rs3026401/*PAX6*  (G→A) |  | hsa-miR-664a-3p |
| rs8192473/CCK  (G→A) | hsa-miR-507 |  |
| ***Proxy SNPs*** |  |  |
| rs2293577/*SLC39A13*  (C→T) |  | hsa-miR-508-5p/hsa-miR-665 |
| rs206942/*NUDT3*/*RPS10-NUDT3*  (T→C) | hsa-miR-490-5p |  |
| rs11030100/*BDNF/BDNF-AS*  (G→T) | hsa-miR-1264 |  |
| rs9909/*NUP160*  (C→G) | hsa-**let-7**f-2-3p |  |
| rs14810/*KCTD15*  (C→G) | hsa-miR-650/hsa-miR-1275 | hsa-miR-486-3p |
| rs1032524 /*NAV1*  (T→C) | hsa-miR-493-5p |  |
| rs2644128 /*NAV1*  (C→G) |  | hsa-miR-449b-3p |
| rs75071083/*GRID1-AS1/ GRID1*  (G→T) |  | hsa-miR-218-1-3p/hsa-miR-218-2-3p |
| rs76636418/*GRID1-AS1/ GRID1*  (T→G) |  | hsa-miR-570-3p |
| rs3812651 /*GRID1-AS1*  (T→G) | hsa-miR-144-3p | hsa-miR-3682 |
| rs7769978/*TFAP2B*  (T→C) |  | hsa-miR-191-5p/ hsa-miR-3148 |
| rs2118404/ *EFR3B*  (C→A) |  | hsa-miR-760 |
| rs1055816 /*PLCD4*  (G→A) |  | hsa-miR-338-3p |
| rs2230115 /*ZNF142*  (G→T) |  | hsa-miR-491-5p/hsa-miR-92b-5p/ hsa-miR-3191-3p |
| rs3770213 /*ZNF142*  (A→T) | **hsa-miR-31-5p** | hsa-miR-485-5p |
| rs11552229 /*ARNT*  (A→G) |  | hsa-**miR-342**-3p |
| rs3752021/ *CRLF3*  (C→G) |  | hsa-**miR-181a**-5p/hsa-miR-181b-5p/hsa-miR-181c-5p/hsa-miR-543 |
| rs762634/ *SULT1A2*  (A→G) | hsa-miR-508-5p |  |
| rs4445669/*CADM1*  (A→G) | hsa-miR-1246 |  |
| rs11558471/*SLC30A8*  (A→G) |  | hsa-miR-3074-5p/hsa-miR-3667-3p |
| rs2645294/*WARS2*  (C→T) |  | hsa-miR-323b-3p |
| rs8024/*IPO9*  (C→A) |  | hsa-**miR-148**b-5p |
| rs8028/*LMOD1*  (A→G) | hsa-miR-888-3p | hsa-miR-185-3p |
| rs951366/*NUCKS1*  (T→C) | hsa-miR-485-5p |  |
| rs823137/*RAB7L1*  (G→A) | hsa-miR-124-5p |  |
| rs861536/*KLC1*  (T→C) |  | hsa-miR-1260a/hsa-miR-1260b/hsa-miR-3620-3p |
| rs2450138/*USP35*  (G→A) | hsa-miR-214-3p | hsa-miR-182-5p/hsa-**miR-196a**-3p |
| rs7154948/*BAG5*  (C→T) |  | hsa-miR-374c-5p |
| rs7693/*BAG5*  (C→T) |  | hsa-miR-708-3p |
| rs1741/ *PDXDC1*  (G→C) |  | hsa-**miR-548**t-5p |
| rs6498540/*PDXDC1*  (A→G) |  | hsa-miR-1260a/hsa-miR-1260b |
| rs2236513/*TOM1L2*  (T→G) | hsa-miR-186-3p |  |
| rs3744115/*TOM1L2*  (G→A) | hsa-miR-185-5p |  |
| rs1108648/*TOM1L2*  (G→A) |  | hsa-miR-181b-3p |
| rs3803455/*LOC145783* (C→T) | hsa-miR-151a-5p | hsa-miR-3173-3p |

Note: MiRNAs in bold have been reported in obesity.
