# Supplementary material for: Computational analyses of obesity associated loci generated by genome-wide association studies
Source: PLoS One. 2018 Jul 2;13(7):e0199987. doi: 10.1371/journal.pone.0199987 (PMC6028139; doi:10.1371/journal.pone.0199987)
Supplement: S5 Table — (DOCX) [file pone.0199987.s005.docx]

**S5 Table. Hub genes in obesity associated genes and their interactions**

| Node 1 | Number of interactions | Node 2 |
| --- | --- | --- |
| *CREB1* | 21 | *BCL2, BDNF, PRKD1, CAMK1D, PRKD3, NTRK2, TAF4, BMP2, TCF7L2, ERBB4, FOXO3, MAP2K3, PPARG, HNF1B, TLR4, MAP2K5, HMGA1, PRKCH, RGS17, IRS1, KAT8* |
| *MC4R* | 18 | *GIPR, LHCGR, CALCR, ADCY3, ADCY9, FTO, TMEM18, BDNF, KCTD15, LEPR, SH2B1, GNPDA2, CCK, SEC16B, BCDIN3D, MTCH2, NTRK2, FAIM2* |
| *PPARG* | 18 | *LPL, RPTOR, SCARB2, TLR4, IRS1, LEPR, TCF7L2, KLF6, BCL2, CDKAL1,CREB1, KCNJ11, IGF2BP2, FTO, SLC30A8, HMGA1, MAP3K12, FOXO3* |
| *TMEM18* | 17 | *KCTD15, GNPDA2, SH2B1, FTO, SEC16B, MC4R, MTCH2, FAIM2, BCDIN3D, GRID1, TFAP2B, TNNI3K, NRXN3, MSRA, BDNF, ETV5,* *DGKG* |
| *FTO* | 17 | *MC4R, TMEM18, CDKAL1, TCF7L2, SLC30A8, IGF2BP2, SH2B1, KCTD15, GNPDA2, HHEX, SEC16B, KCNJ11, MTCH2, PPARG, BCDIN3D, TFAP2B, FAIM2* |
| *BCL2* | 16 | *PMAIP1, CREB1, CTSS, IRS1, PARK2, TOMM40, FOXO3, BDNF, TLR4, PAX5, PPARG, PRKCH, SDC1, MAP2K3, NTRK2, LEPR* |
| *TCF7L2* | 14 | *BTRC, SLC30A8, CDKAL1, IGF2BP2, FTO, KCNJ11, HHEX, GIPR, PPARG, CREB1, BMP2, KCNQ1, CAMK1D, HNF1B* |
| *IRS1* | 13 | *RPTOR, PRKD1, SH2B1, NTRK2, LEPR, BCL2, PPARG, LPL, ATP2A1, FOXO3, CREB1, SCARB2, DUSP9* |
| *SH2B1* | 12 | *NTRK2, TMEM18, KCTD15, GNPDA2, SEC16B, MTCH2, FTO, MC4R, IRS1, NPC1, BCDIN3D, FAIM2* |
| *KCNJ11* | 11 | *HSD17B12, TCF7L2, SLC30A8, CDKAL1, IGF2BP2, KCNQ1, HHEX, FTO, PPARG, HNF1B, LEPR* |
| *SEC16B* | 11 | *TMEM18, KCTD15, GNPDA2, BCDIN3D, SH2B1, MTCH2, FTO, MC4R, FAIM2, TFAP2B, TNNI3K* |
| *SLC30A8* | 11 | *CDKAL1, IGF2BP2, TCF7L2, HHEX, FTO, KCNJ11, SLC39A8, CAMK1D, KCNQ1, PPARG, HNF1B* |
| *BCDIN3D* | 10 | *KCTD15, SEC16B, SH2B1, FAIM2, GNPDA2, DGKG, MTCH2, FTO, MC4R, TMEM18* |
| *BTRC* | 10 | *TCF7L2, PARK2, HUWE1, HERC4, ASB4, PSME4, RBBP6, HIVEP1, FOXO3, USP37* |
| *CDKAL1* | 10 | *SLC30A8, IGF2BP2, TCF7L2, HHEX, FTO, KCNJ11, CAMK1D, KCNQ1, PPARG, HNF1B* |
| *FOXO3* | 10 | *TNRC6B, MAPKAPK5, RPTOR, BTRC, PMAIP1, BCL2, IRS1, ETS2, CREB1, PPARG* |
| *GNPDA2* | 10 | *TMEM18, KCTD15, SEC16B, SH2B1, MTCH2, BCDIN3D, FTO, MC4R, FAIM2, TFAP2B* |
| *IGF2BP2* | 10 | *CDKAL1, SLC30A8, TCF7L2, HHEX, FTO, KCNJ11, CAMK1D, KCNQ1, PPARG, HNF1B* |
| *MTCH2* | 10 | *TMEM18, KCTD15, GNPDA2, SH2B1, BCDIN3D, SEC16B, FTO, TOMM40, MC4R, FAIM2* |
| *KCTD15* | 10 | *TFAP2B, TMEM18, GNPDA2, SH2B1, SEC16B, BCDIN3D, MTCH2, MC4R, FTO, FAIM2* |
| *RIT2* | 10 | *RIT1, MDFIC, KNTC1, ETV5, MAP2K5, DMRTA1, GNAT2, LRFN2, LINGO2, KAT8* |
