# Supplementary material for: Computational analyses of obesity associated loci generated by genome-wide association studies
Source: PLoS One. 2018 Jul 2;13(7):e0199987. doi: 10.1371/journal.pone.0199987 (PMC6028139; doi:10.1371/journal.pone.0199987)
Supplement: S7 Table — (DOCX) [file pone.0199987.s007.docx]

**S7 Table.** **Enriched signaling pathways of obesity GWAS genes**

| **Pathway ID** | **Pathway description** | **Count in gene set** | **FDR** | **Official gene symbols** |
| --- | --- | --- | --- | --- |
| 4514 | Cell adhesion molecules | 10 | 0.00371 | *CADM1*,*SDC1*,*HLA-DQA1*,*HLA-DRA*,*HLA-DRB1*,*HLA-DRB5*,*NCAM2*,*NRXN1*,*NRXN2*,*NTNG1* |
| 5321 | Inflammatory bowel disease | 7 | 0.00371 | *HLA-DQA1*,*HLA-DRA*,*HLA-DRB1*,*HLA-DRB5*,*MAF*,*TLR4*, *IFNGR1* |
| 5145 | Toxoplasmosis | 8 | 0.018 | *BCL2*,*IFNGR1*,*HLA-DQA1*,*HLA-DRA*,*HLA-DRB1*,*MAP2K3*,*HLA-DRB5*,*TLR4* |
| 4612 | Antigen processing and presentation | 6 | 0.0201 | *CREB1*,*CTSS*,*HLA-DQA1*,*HLA-DRA*,*HLA-DRB1*,*HLA-DRB5* |
| 4950 | Maturity onset diabetes of the young | 4 | 0.0201 | *HHEX*,*HNF1B*,*HNF4G*,*PAX6* |
| 5140 | Leishmaniasis | 6 | 0.0201 | *HLA-DQA1*,*HLA-DRA*,*HLA-DRB1*,*HLA-DRB5*,*IFNGR1*, *LR4* |
| 5310 | Asthma | 4 | 0.0247 | *HLA-DQA1*,*HLA-DRA*,*HLA-DRB1*,*HLA-DRB5* |
| 5152 | Tuberculosis | 9 | 0.0252 | *BCL2*,*CREB1*,*CTSS*,*TLR4*,*HLA-DQA1*,*HLA-DRA*,*HLA-DRB1*,*HLA-DRB5*,*IFNGR1* |
| 5416 | Viral myocarditis | 5 | 0.0333 | *HLA-DQA1*,*HLA-DRA*,*HLA-DRB1*,*HLA-DRB5*,*DMD* |
| 4722 | Neurotrophin signaling pathway | 7 | 0.0334 | *BCL2*,*BDNF*,*FOXO3*,*IRS1*,*MAP2K5*,*NTRK2*,*SH2B1* |
| 5330 | Allograft rejection | 4 | 0.0334 | *HLA-DQA1*,*HLA-DRA*,*HLA-DRB1*,*HLA-DRB5* |
| 5032 | Morphine addiction | 6 | 0.0344 | *ADCY3*,*ADCY9*,*GABRA5*,*GABRB3*,*GABRG1*,*PDE1C* |
| 4972 | Pancreatic secretion | 6 | 0.0351 | *ADCY3*,*ADCY9*,*ATP2A1*,*KCNMA1*,*RAB27B*, *KCNQ1* |
| 5332 | Graft-versus-host disease | 4 | 0.0351 | *HLA-DQA1*,*HLA-DRA*,*HLA-DRB1*,*HLA-DRB5* |
| 4940 | Type I diabetes mellitus | 4 | 0.0451 | *HLA-DQA1*,*HLA-DRA*,*HLA-DRB1*,*HLA-DRB5* |
